# Insights from a Cross-Sectional Study on Knowledge, Attitudes and Behaviors Concerning Antibiotic Use in a Large Metropolitan Area: Implications for Public Health and Policy Interventions

**DOI:** 10.3390/antibiotics12101476

**Published:** 2023-09-22

**Authors:** Francesca Pennino, Maria Luisa Maccauro, Michele Sorrentino, Mariagiovanna Gioia, Simonetta Riello, Giuseppe Messineo, Carmela Di Rosa, Paolo Montuori, Maria Triassi, Antonio Nardone

**Affiliations:** Department of Public Health, “Federico II” University, Via Sergio Pansini nº 5, 80131 Naples, Italy

**Keywords:** antibiotics, KAP model, knowledge, attitude, behaviors, questionnaire, multiple linear regression

## Abstract

The overuse and inappropriate use of antibiotics pose a grave threat to public health, contributing significantly to the accelerated development of antimicrobial resistance (AMR) and increased rates of morbidity and mortality, making it a leading cause of death globally. To examine the relationship between demographic variables and knowledge, attitudes, and behaviors concerning antibiotic use, a survey-based cross-sectional study was conducted involving 1158 individuals. The questionnaire included two sections: in the first section, participants’ socio-demographic characteristics were analyzed; the second investigated knowledge, attitudes, and behaviors concerning antibiotics utilization using a total of 36 questions. Descriptive statistics were used, and then a multiple linear regression analysis (MLRA) using three models was carried out. In Model I, knowledge about antibiotics exhibited correlations with smoking habits and educational attainment. In Model II, attitudes were significantly associated with gender, smoking habits, age, education, relationship status, and knowledge. In Model III, behaviors related to antibiotics were correlated with educational attainment, having children, knowledge, and attitudes. Implementing tailored public health programs could be a cost-effective intervention to enhance behaviors associated with antibiotic use in the general population. This study offers valuable insights into the determinants of knowledge, attitudes, and behaviors regarding antibiotics in the general population.

## 1. Introduction

Overuse and inappropriate use of antibiotics pose a serious threat to public health, animal health, environmental health, and global economic development [1,2,3,4,5], but also contribute significantly to the accelerated development of antimicrobial resistance (AMR) [1,6,7].

The overuse of antibiotics, exacerbated by the impact of the COVID-19 pandemic [8], presents a multifaceted issue with significant implications. This includes the accelerated transmission of diseases, prolonged hospitalization periods, escalated healthcare expenses, elevated rates of treatment ineffectiveness, as well as increased morbidity and mortality rates [9,10,11,12].

Moreover, antibiotic overuse is a leading cause of death worldwide, particularly in low- and middle-income countries [13,14,15,16,17]. AMR is associated with approximately 4.95 million deaths [18], and if left unaddressed, this number could rise to an estimated 10 million deaths annually by 2050 [19].

The inappropriate use of antibiotics is often associated with inadequate knowledge and awareness regarding their proper use, both among healthcare providers and patients [20,21]. This issue is further exacerbated by the widespread availability of over-the-counter drugs that do not require a prescription, which contributes to self-medication practices [22].

To effectively tackle the challenge of antibiotic overuse, it is crucial to raise awareness among healthcare providers and the general public about the dangers of antibiotic misuse and the importance of responsible antibiotic use [19,23].

Numerous ongoing studies utilize the KAP model to explore the underlying reasons for inappropriate antibiotic use. These studies focus on assessing knowledge about antibiotic use, understanding attitudes towards antibiotics, and examining practices related to antibiotic consumption.

For instance, several studies have focused on measuring the knowledge, attitudes, and behaviors of medical students towards antibiotics. Although some studies show encouraging results, many highlight a lack of knowledge regarding the importance of correct antibiotic use and prescription [24,25,26,27,28,29,30,31].

Numerous studies have investigated the knowledge and attitudes of the general population in various countries, revealing notable differences: some studies have indicated low levels of knowledge regarding the proper use of antibiotics and a high prevalence of self-medication, raising significant concerns about antibiotic practices [28,32,33,34,35,36]. Other studies have also highlighted differences between rural and urban areas [37,38]. However, there were instances wherein individuals demonstrated good knowledge and practice in using antibiotics, but their overall attitude towards antibiotic use was insufficient [39].

In Italy, there is limited research on the knowledge and attitudes of the general population regarding antimicrobial drugs. Only one study has been conducted, and it highlights a concerning lack of knowledge among the population [39]; in fact, Italy has been identified as one of the worst-performing nations in Europe in terms of antibiotic use [40,41], with a higher prevalence of antibiotic utilization compared to other European nations, characterized by substantial consumption of broad-spectrum medications [42,43].

The objective of this research is to assess individuals’ understanding, perspectives, and practices pertaining to the proper utilization of antibiotics. By accomplishing this, it becomes possible to identify effective interventions and strategies that can help address the problem of antibiotic resistance. Through comprehensive evaluation, valuable insights can be gained, leading to the development of targeted initiatives aimed at promoting responsible antibiotic use and combatting the rise of antibiotic resistance.

## 2. Methods

### 2.1. Setting and Sample

This observational cross-sectional research was carried out from May 2022 to August 2022 by administering questionnaires to the adults of the metropolitan city of Naples in the South of Italy, with a population of 909,048 [44]. Some 1560 subjects among university, working places and, community centers were selected to participate at the study. Among those, 1158 agreed to participate to the survey, returning a complete questionnaire, with a response rate of 74.23%. Participants had to be at least 18 years old and live in the Naples metropolitan region to be eligible for the research. In order to obtain a representative sample within a margin of error of 3%, and a confidence interval of 95%, a Slovin’s formula was used to evaluate the required sample size. Specifically, the number of subjects to be recruited had to be 1523, which, accounting for a 30% non-response rate, had to be 1066.

### 2.2. Procedures

The questionnaire was submitted to the respondents from Monday to Friday between 10:00 a.m. and 8:00 p.m. in order to prevent oversampling nonworking people. Before beginning the questionnaire, each respondent was informed that the study was being undertaken on behalf of the “Federico II” University of Naples. Furthermore, participants were informed about the nature, scope, and methods of the study. Additionally, the respondents were informed that their involvement was entirely voluntary, that all information gathered would be handled anonymously and confidentially, and that they might discontinue their participation at any moment without explanation. Before proceeding with the interview, verbal informed consent was obtained. There was no incentive to take part or finish the survey. The study was conducted in accordance with the Helsinki Declaration, and ethical approval was acquired in accordance with local regulations.

### 2.3. Data Collection

The survey was developed by a commission of health professionals. The appropriateness and usefulness of questions was analyzed during a meeting, and questions considered not appropriate or useful for the study objectives were either removed or replaced [45,46]. According to Andrade et al., the questions were carefully crafted. As a result, questions too easy or with obvious answers were avoided. Similarly, questions that are too complex or that may mean various things to different individuals, have difficult vocabulary or ideas, employ technical terminology or colloquial phrases, are long and difficult to grasp, cover more than one subject in the same question, or involve double negatives were excluded. The options for the answers had to be created with caution to prevent respondents from being forced into selecting an option that they may not truly endorse [47]. Before submitting the survey, a beta-test with trusted subjects was performed to assess the ease-of-comprehension and coherence of the questionnaire. Particularly, a pilot study was performed on 10 individuals in order to test the participants’ understanding of the questionnaire items, the results of which were not taken into consideration for the study. In the first section of the questionnaire, the participants’ socio-demographic characteristics and other health-related information, such as gender, age, marital status, level of education, occupation, partner’s occupation, and number of children, were indicated. The second section examined knowledge, attitudes, and behaviors concerning antibiotic utilization for a total of 36 questions. A three-point Likert scale with options for “agree”, “uncertain” and “disagree” was used in order to assess the knowledge and attitudes, while behaviors were estimated using a four-answer style of “never”, “sometimes”, “often” and “yes/always”.

### 2.4. Statistical Analysis

The collected data has been processed using the STATA MP v14.0 statistical software program (College Station, TX, USA). The analysis consists of a first part, in which descriptive statistics were used, and a second part, in which a multiple linear regression analysis (MLRA) was conducted using dependent and independent variables to evaluate their correlation. MLRA allows us to evaluate the statistical significance of the regression model within values of *p* < 0.05, and to estimate significance of both the beta coefficient and coefficient of determination in order to determine how the results of present study vary in relation to independent variables.

Three models of MLRA were considered:

(1) Knowledge about the proper use of antibiotics (Model 1);
(2) Attitudes on the use of antibiotics (Model 2);
(3) Behaviors related to the use of antibiotics in daily life (Model 3).

The dependent variables (knowledge, attitudes, and behaviors) were calculated by adding the scores from the respective questions (questions with inverted responses were encoded in reverse). Gender (1 = male, 2 = female); age, in years; level of education (1 = primary school, 2 = middle school, 3 = high school, 4 = university degree); single (1 = single; 2 = in a relationship); career, and smoking (1 = smoker, 2 = non-smoker) were all included as independent variables in all models. In Model 2, we included knowledge in the independent variables, and in Model 3, we included knowledge and attitudes in the independent variables. In the study, attitudes and knowledge were treated as indices rather than scales, implying that each observable variable (A1, …, A12 and K1, …, K12) is critical in causing the related latent variables (attitudes and knowledge). All statistical tests were two-tailed, and results were statistically significant if the *p*-values were less than or equal to 0.05.

## 3. Results

Table 1 shows the characteristics of the population studied, which was found to have a mean age of 34.76 ± 19.00 years old, and the largest group in the distribution was 28.14 ± 2.57 years old, accounting for 41.97%, followed by those under 25 years old, who represented 32.64% of the total population.

Table 2 presents the respondents’ knowledge about the use of antibiotics. According to the table, the majority of the participants, 89.37%, knew that antibiotics can be used to treat infections caused by bacteria, but only 46.20% were aware that antibiotics are ineffective for viral infections. The study also revealed that 83.94% of the participants knew that antibiotics could have side effects, but 37.30% agreed with the use of antibiotics for pain relief. Interestingly, most of the participants, 66.49%, agreed that antibiotics should not be discontinued after symptoms disappear. The results also showed that 71.24% of respondents knew that frequent use of antibiotics can reduce their effectiveness, while 77.37% were not aware that there are bacteria that are resistant to all antibiotics. In addition, 59.50% of the participants understood that antibiotic resistance indicates the survival of bacteria against antibiotics, and 46.98% knew that skipping one or two doses of antibiotics could contribute to antibiotic resistance.

The attitudes of the respondents towards the use of antibiotics are presented in Table 3. The majority of the participants, 76.77%, disagreed with the statement that antibiotics never hurt and that antibiotics should be taken immediately when experiencing a sore throat (79.00%). Only a small percentage of the respondents, 14.43%, agreed that consulting a doctor is unnecessary. When asked about taking antibiotics from friends and relatives for treating the same symptom, 58.72% of the participants disagreed that it was possible to do so. However, over 89% of the respondents knew that the expiration date is crucial for the consumption of antibiotics. Only a small percentage of the participants, 9.24%, agreed that skipping one or two doses of antibiotics is irrelevant, and 10.29% agreed that one antibiotic is as good as another.

In Table 4, the behaviors of respondents regarding the use of antibiotics are presented. The results indicate that only 27.03% of the respondents answered “never” when asked if they take antibiotics when they have the flu. A majority of the respondents, 90.93%, reported taking antibiotics for sore throats. When asked if they take antibiotics only after medical consultation, only 0.52% of the participants answered “never”. Only 16.58% of the respondents stated that they do not use the same antibiotic for the same symptom. Unfortunately, 49.65% of the participants reported that they still tend to stop taking antibiotics when their symptoms disappear. The majority of the interviewees, 83.25%, reported checking the drug expiration date before taking antibiotics. However, 76.42% of the participants reported taking antibiotics without doing an antibiogram before.

The findings of a multiple logistic regression analysis (MLRA) using three models are shown in Table 5. With Model I, there was a correlation between knowledge (used as an independent variable) and education and smoking behaviors. Model II demonstrates a statistically significant link between attitudes and age, gender, education, smoking behaviors, and knowledge. Model III revealed a statistically significant relationship between actions and marital status, children, knowledge, and attitudes.

**Figure 1:**
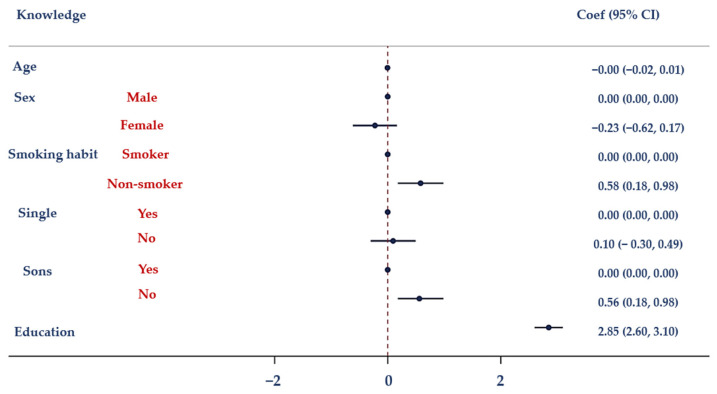
Results of the MLRA. Correlation between knowledge regarding antibiotics and age, sex, smoking habits, marital status, having children and education.

**Figure 2:**
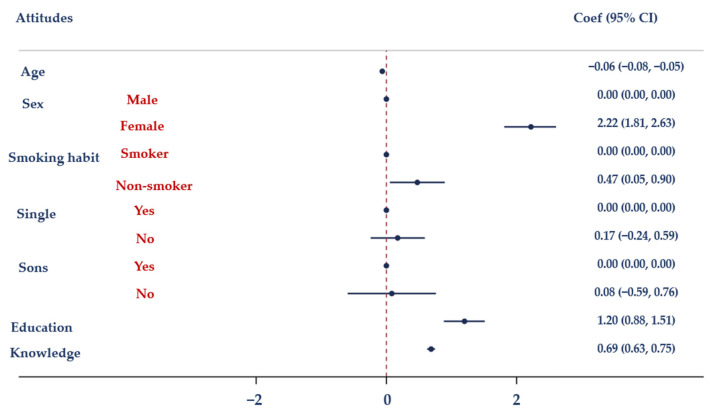
Results of the MLRA. Correlation between attitudes regarding the proper use of antibiotics and age, sex, smoking habits, marital status, having children and education.

**Figure 3:**
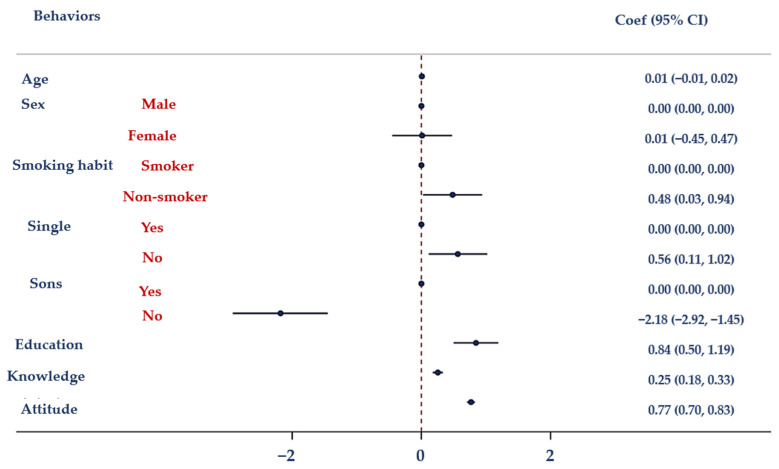
Results of the MLRA. Correlation between behaviors regarding the proper use of antibiotics and age, sex, smoking habits, marital status, having children and education.

Figure 1 depicts the association between antibiotic knowledge and demographic characteristics (age, gender, smoking habits, marital status, having children, and educational level). Moreover, the association between attitudes towards the proper use of antibiotics and demographic factors (including age, sex, smoking habits, marital status, having children, and education attainment) is displayed in Figure 2. Figure 3 illustrates the relationship between antibiotic-related behaviors and demographic characteristics such as age, sex, smoking habits, marital status, having children, and education level.

## 4. Discussions

This study underscores the critical importance of addressing antibiotic overuse and misuse to combat antimicrobial resistance and its global health implications. The findings, derived from a comprehensive survey-based study involving 1158 individuals, highlight the intricate relationship between demographic variables, knowledge, attitudes, and behaviors related to antibiotic use. Therefore, this research provides a foundation for implementing effective public health programs aimed at improving antibiotic-related behaviors in the general population, ultimately contributing to a healthier and more informed society.

In the results presented in Table 5, Model I revealed an association between education level and knowledge about antibiotics. These findings are coherent with several previous studies which found that college graduates tend to have higher levels of knowledge about antibiotics compared to those with less education [33,34,39,44]. The positive correlation observed between education level and knowledge about antibiotics can be attributed to various factors, including improved health literacy, greater exposure to scientific information, and access to academic resources like publications and peer-reviewed journals [48,49]. However, it is essential to recognize that educational opportunities are not equally distributed globally, and individuals from socio-economically disadvantaged backgrounds may face limited access to education, which can negatively affect their understanding of antibiotics. This knowledge gap can contribute to the overuse and misuse of antibiotics, thereby fostering the development of AMR.

Furthermore, this research has consistently shown a positive correlation between non-smoking habits and knowledge regarding antibiotics. Individuals who do not smoke exhibit greater awareness and understanding of health risks associated with antibiotic use [50,51]. However, since this finding could suggest the possible benefits of promoting non-smoking behaviors in relation to antibiotic knowledge, there remains a gap in research regarding the underlying mechanisms behind this observed correlation and the potential causality between non-smoking habits and knowledge about antibiotic use. Further investigations are necessary to delve into the complex relationship between smoking habits, attitudes toward antibiotics, and health outcomes. Future research efforts should aim to address these gaps in understanding.

The second piece of evidence, as shown in Table 5, is provided by Model II, which highlights a correlation between non-smoking habits and attitudes toward antibiotics, probably due to more respiratory illnesses occurring among the smoking group. The influence of smoking habits on the usage of antibiotics has been well documented [52]. This finding highlights the significance of taking smoking behavior into account when studying attitudes and behaviors regarding antibiotic use. Acknowledging the impact of smoking habits on antibiotic attitudes allows for the development of targeted interventions and educational campaigns aimed at promoting responsible antibiotic use among individuals who smoke. By addressing this specific population, we can effectively contribute to the reduction of antibiotic overuse and the preservation of antibiotic effectiveness.

Consistent with previous research [33,48], this study reveals an association between being a woman and attitudes regarding antibiotics. Several factors may contribute to this finding. In fact, women tend to exhibit a lower inclination for risk-taking, and tend to prioritize their health [53]. Furthermore, women generally have more frequent contact with healthcare providers throughout their lives [54,55]. These factors contribute to a heightened focus on health-related matters, including the proper use of antibiotics. It is important to note that the findings of our study differ from those of a survey conducted in Bangladesh, which found that males were more knowledgeable about antibiotics than females [56]. This disparity may be attributed to cultural or societal differences in the specific region studied.

Another result of this study was the association between older age and attitudes regarding antibiotics, which is consistent with previous research [33,39,57]. This relationship can be attributed to the fact that younger individuals have limited experience with antibiotics, which may contribute to a lower level of understanding in these areas [58]. Nowadays, the Internet is becoming a more popular source of health-related information, and social media may be utilized to encourage antibiotic prudence, particularly among younger people. However, in order to encourage proper use of the Internet for antibiotic-related information seeking in the general community, health organizations must include social media in their communication plans, according to Zucco et al., 2018, and Parveen et al., 2022 [59,60].

Overall, this study highlights the importance of considering age as a factor when examining people’s attitudes towards antibiotics, as this can help healthcare professionals to better understand how to communicate with different age groups about appropriate use of antibiotics. Previous research has demonstrated that evidence-based interactive health literacy programs designed specifically for older adults have been effective in enhancing knowledge and skills related to health decision-making [61].

Furthermore, the study revealed a noteworthy association between knowledge and attitude, suggesting that knowledge regarding antibiotics may contribute to the development of positive attitudes. This finding is consistent with previous studies in which the relationship between attitudes and knowledge has been assessed [38,62]. Possessing accurate and reliable information about antibiotics is essential for making well-informed decisions about the appropriate use of antibiotics and cultivating positive attitudes towards responsible antibiotic use.

In Table 5, Model III, the findings indicate a correlation between having children and exhibiting better behaviors. This is significant because parents are the primary individuals responsible for making healthcare decisions for their children [63]. Therefore, targeting parents may be a crucial approach to promote responsible antibiotic use and prevent AMR, ultimately leading to a significant decrease.

Furthermore, this study identified a relationship between individuals’ education levels and their behavior regarding the use of antibiotics. This finding is not surprising, as previous research has also reported a similar connection between education and antibiotic use [64]. It is possible that individuals with a higher level of education have a better understanding of the risks associated with antibiotic misuse, and are therefore, more likely to exhibit responsible behaviors.

Additionally, this study revealed a noteworthy association between a higher level of knowledge and attitudes about antibiotics and the adoption of healthy behaviors, in contrast with other studies [33,38]. Our finding suggests that educating individuals about the risks associated with antibiotic misuse and providing them with proper information can significantly contribute to the promotion of responsible antibiotic use and prevent the development of antimicrobial resistance, which is particularly noteworthy, as it contradicts the results of previous studies that reported different outcomes.

## 5. Limitation

The findings of this study should be interpreted with caution due to several limitations that need to be considered. One major limitation is that the study relied on self-reported behaviors obtained through questionnaires not validated previously, which could have led to social desirability bias, in addition to the fact that answers may be subjective. However, the study attempted to address this issue by ensuring participant anonymity and confidentiality. Another potential limitation is the possibility of selection bias, because the sample selection was not random, as certain individuals or groups may have been excluded from the study; thus, the results could be affected by reliability problems. Confounding bias is also a possibility, if there were other variables that influenced the results but were not accounted for in the analysis. Moreover, the study’s small sample size relative to the population of Naples limits the generalizability of the findings. Furthermore, while the use of a KAP-based questionnaire was helpful in capturing knowledge, attitudes, and practices related to antibiotics, it may not have captured all the factors that influence antibiotic-related beliefs and behaviors. Furthermore, while every effort was made to ensure the accuracy and reliability of our data, it is essential to acknowledge the potential limitations of this study. First, the sample size, though representative of the target population, may have limited the generalizability of our findings. Second, the absence of a long-term follow-up restricts our ability to draw conclusions about the sustainability of the observed effects. Thirdly, no statistically significant differences were found in the subgroups of the sample; however, these results may be influenced by the numerosity of such subgroups. Lastly, although an adjusted analysis would have provided a more robust approach to the methodology of this study, our research design and resources constrain us from conducting further analysis. These limitations should be considered when interpreting the results and designing future research endeavors in this area.

## 6. Policies

The appropriate use of antibiotics is a pressing concern that demands immediate attention. The findings of this study underscore the vital role of knowledge and attitudes in promoting the proper use of antibiotics and reducing the spread of AMR. It is important to recognize that misconceptions and preconceived notions regarding antibiotics and their effects persist among the general public, indicating the need for the continuous evaluation and improvement of such campaigns to ensure their effectiveness [65,66].

To effectively address the issue of antibiotic overuse and promote appropriate antibiotic use, the implementation of comprehensive antibiotic stewardship programs is crucial, aiming to mitigate overuse and encourage responsible practices [67]. Consistent reinforcement and multifaceted strategies, applied over time, can help counteract misinformation on antibiotics and their irrational use [32], focusing on enhancing knowledge about the appropriate use and prescription of antimicrobial drugs [68].

Various channels, including social media, television, and newspapers, as well as the engagement of healthcare professionals, can be used effectively to distribute information about the proper use of antibiotics [32,62].

In conclusion, promoting appropriate antibiotic use requires a multifaceted approach that includes robust antibiotic stewardship programs, continuous evaluation of national campaigns, and the utilization of insights from research and activities aimed at both the general public and healthcare professionals. By implementing these strategies, we can strive to mitigate antibiotic overuse, combat antibiotic resistance, and safeguard the effectiveness of these essential medications. Ultimately, these efforts can contribute to reducing the overall burden of resistant microbes and AMR, promoting the well-being of humans and all other living beings.

## 7. Conclusions

This research provided valuable insights into the impact of demographic factors, educational attainment, knowledge, attitudes, and behaviors on antibiotic use in the general population. However, it is important to acknowledge and address the limitations inherent in the study’s cross-sectional design when interpreting the findings, as the selected respondents may not fully represent the wider population. Additionally, relying on self-reported behaviors may be susceptible to limitations related to memory and social desirability bias.

The research emphasizes the interplay between knowledge, attitudes, and behaviors, and reveals that knowledge plays a crucial role in influencing attitudes and behaviors related to antibiotic use, as evidenced by the fact that individuals with greater knowledge exhibit more positive attitudes and engage in more responsible behavior. These findings underscore the importance of addressing misconceptions and promoting accurate knowledge about antibiotics to drive positive behavioral change. Furthermore, specific demographic factors were identified as influencers of attitudes and behaviors related to antibiotic use.

These significant findings, which were the intended aim of the study, have noteworthy implications for public health policies, providing valuable insights for targeted interventions and campaigns, recognizing the need to tailor educational initiatives to different demographic groups in order to promote accurate knowledge and to encourage positive behaviors. These behaviors will play a crucial role in reducing antibiotic overuse and preserving the effectiveness of antibiotics, thereby mitigating the threat of AMR. By acting now, we can ensure the continued efficacy of antibiotics for future generations.

## Figures and Tables

**Table 1:** Study population characteristics and scores of knowledge, attitudes and behaviour.

Study Population	N	Percentage
Sex	(1158)	
Male	479	41.36
Female	679	58.64
Age		
<25	378	32.64
25–35	486	41.97
36–55	70	6.05
>55	224	19.34
Education		
Primary school	57	4.92
Middle school	168	14.51
High school	579	50.00
University degree	354	30.57
Sons		
Yes	235	20.30
No	923	79.70
Smoking habits		
Yes	520	44.90
No	638	55.10
Marital Status		
Single	535	46.20
In a relationship	623	53.80

**Table 2:** Knowledge of respondents regarding antibiotics.

N.	Statement (Variables)	Agree (%)	Uncertain (%)	Disagree (%)
K1	Antibiotics are effective against bacteria	89.37	9.33	1.30
K2	Antibiotics are effective against viruses.	37.82	15.98	46.20
K3	Antibiotics have side effects.	83.94	13.64	2.42
K4	Antibiotics are useful for pain relief.	37.30	13.30	49.40
K5	The antibiotic should be stopped when symptoms disappear.	19.86	13.65	66.49
K6	Frequent use of antibiotics reduces their effectiveness.	71.24	17.70	11.06
K7	There are bacteria that are resistant to all antibiotics.	22.63	49.48	27.89
K8	The antibiogram evaluates antibiotic sensitivity.	52.50	41.20	6.30
K9	Antibiotic resistance indicates the survival of bacteria against antibiotics.	59.50	28.41	12.09
K10	Skipping one or two doses of antibiotic may contribute to antibiotic-resistance.	46.98	28.93	24.09
K11	The sale of antibiotics without a prescription is prohibited.	55.53	17.96	26.51
K12	Fascia A antibiotics are only used in hospitals.	15.55	59.84	24.61

**Table 3:** Attitude of respondents toward proper use of antibiotics.

N.	Statement (Variables)	Agree (%)	Uncertain (%)	Disagree (%)
A1	It is essential to always carry a drug.	45.51	15.37	39.12
A2	One antibiotic is as good as another.	10.79	11.92	77.29
A3	Antibiotics do not cause harm.	7.25	15.98	76.77
A4	You should take antibiotics immediately when you have a sore throat.	11.31	9.24	79.45
A5	It is useless to have a blood test if you feel good.	10.54	4.66	84.80
A6	Often, consulting your doctor is superfluous.	14.43	13.64	71.93
A7	It is preferable to take drugs that you already have at home.	23.75	14.85	61.40
A8	It is possible to take antibiotics from friends and relatives, if used to treat the same symptom.	23.84	17.44	58.72
A9	Reading the package’s leaflet is useless.	19.17	2.33	78.50
A10	The expiry of a drug is not relevant.	5.10	5.35	89.55
A11	Antibiotics should not be taken without an antibiogram.	17.27	42.57	40.16
A12	Skipping one or two doses of antibiotics is irrelevant.	9.24	10.54	80.22

**Table 4:** Behaviours of respondents toward proper use of antibiotics.

N.	Questions	Yes/Always (%)	Often (%)	Sometimes (%)	Never (%)
B1	Do you take antibiotics when you have the flu?	13.73	6.30	52.94	27.03
B2	Do you take antibiotics when you have a sore throat?	31.52	49.05	10.36	9.07
B3	Do you take antibiotics to treat urinary infections?	21.16	12.61	32.72	33.51
B4	Do you only take antibiotics after medical consultation?	52.16	14.94	32.38	0.52
B5	Do you take the same antibiotic to treat the same symptom?	23.23	23.49	36.70	16.58
B6	Do you stop the antibiotic if you forget to take a dose?	5.96	9.15	8.81	76.08
B7	Do you stop the antibiotic if your symptoms disappear?	13.99	7.86	28.50	49.65
B8	Do you usually carry antibiotics when you travel?	32.47	18.83	22.19	26.51
B9	Do you read the drug’s leaflet?	45.94	19.43	20.29	14.34
B10	Do you read the drug’s expiration date?	83.25	7.51	7.17	2.07
B11	Do you take antibiotics without doing an antibiogram?	76.42	2.42	14.34	6.82
B12	Have you happened to miss one or two doses of antibiotics?	37.39	4.84	23.49	34.28

**Table 5:** Results of the linear multiple regression (MLRA).

	Coefficients Not Standardized		Coefficients Standardized			
	b	Standard Error	t	95% Conf. Interval		*p*-Value
Model I—Dependent variable: Knowledge						
Prob > F = 0.000	R-squared = 0.4744			Root-MSE = 3.4311		
Age	−0.004	0.008	−0.50	−0.019	0.011	0.615
Sex	−0.225	0.199	−0.13	−0.616	0.165	0.258
Marital status	0.096	0.203	0.47	−0.303	0.495	0.636
Children	0.561	0.331	1.70	−0.88	1.21	0.090
Smoking habits	0.582	0.205	2.84	0.180	0.985	0.005
Education	2.85	0.128	22.23	2.60	3.10	0.000
Model II—Dependent variable: Attitudes						
Prob > F = 0.000	R-squared = 0.4744			Root-MSE = 3.4311		
Age	−0.063	0.008	−7.96	−0.079	−0.048	0.000
Sex	2.22	0.208	10.68	1.81	2.63	0.000
Marital status	0.173	0.212	0.82	−0.242	0.590	0.414
Children	0.083	0.345	0.24	−0.595	0.761	0.810
Smoking habits	0.475	0.215	2.21	0.053	0.897	0.027
Education	1.20	0.160	7.49	0.883	1.51	0.000
Knowledge	0.687	0.031	22.33	0.626	0.747	0.000
Model III—Dependent variable: Behaviour						
Prob > F = 0.000	R-squared = 0.4744			Root-MSE = 3.4311		
Age	0.007	0.088	0.76	−0.011	0.024	0.446
Sex	0.011	0.236	0.05	−0.452	0.474	0.962
Marital status	0.563	0.230	2.45	0.112	1.02	0.015
Children	−2.18	0.374	−5.84	−2.92	−1.45	0.000
Smoking habits	0.483	0.233	2.07	0.025	0.94	0.039
Education	0.845	0.177	4.76	0.497	1.19	0.000
Knowledge	0.254	0.040	6.37	0.176	0.332	0.000
Attitude	0.768	0.032	24.03	0.705	0.830	0.000

## Data Availability

The data that support the findings of this study are available upon reasonable request from the corresponding author. The data are not publicly available due to privacy or ethical restrictions.

## References

[1] Bell B.G., Schellevis F., Stobberingh E., Goossens H., Pringle M. (2014). A systematic review and meta-analysis of the effects of antibiotic consumption on antibiotic resistance. BMC Infect. Dis..

[2] Costelloe C., Metcalfe C., Lovering A., Mant D., Hay A.D. (2010). Effect of antibioticprescribing in primary care on antimicrobialresistance in individualpatients: Systematic review and meta-analysis. BMJ.

[3] Forslund K., Sunagawa S., Kultima J.R., Mende D.R., Arumugam M., Typas A., Bork P. (2010). Country-specific antibiotic use practices impact the human gut resistome. Genome Res..

[4] World Health Organization (WHO) Global Antimicrobialresistance and Use Surveillance System (GLASS) Report: 2022. December 2022. https://www.who.int/publications/i/item/9789240062702.

[5] Zhang Y., Steinman M.A., Kaplan C.M. (2012). Geographic Variation in Outpatient Antibiotic Prescribing Among Older Adults. Arch. Intern. Med..

[6] Shallcross L.J., Howard S.J., Fowler T., Davies S.C. (2015). Tackling the threat of antimicrobial resistance: From policy to sustainable action. Philos. Trans. R. Soc. B Biol. Sci..

[7] Ruiz J. (2021). Antimicrobial Resistance, from bench-to-publicside. Microbes Infect. Chemother..

[8] World Health Organization (WHO) Preventing the COVID-19 Pandemic from Causing an Antibiotic Resistance Catastrophe. November 2020. https://www.who.int/europe/news/item/18-11-2020-preventing-the-covid-19-pandemic-from-causing-an-antibiotic-resistance-catastrophe.

[9] Cantón R., Akova M., Langfeld K., Torumkuney D. (2022). Relevance of the Consensus Principles for Appropriate Antibiotic Prescribing in 2022. J. Antimicrob. Chemother..

[10] Cassini A., Högberg L.D., Plachouras D., Quattrocchi A., Hoxha A., Simonsen G.S., Colomb-Cotinat M., Kretzschmar M.E., Devleesschauwer B., Cecchini M. (2019). Attributable deaths and disability-adjusted life-years caused by infections with antibiotic-resistant bacteria in the EU and the European Economic Area in 2015: A population-level modelling analysis. Lancet Infect. Dis..

[11] Murray C.J.L., Ikuta K.S., Sharara F., Swetschinski L., Robles Aguilar G., Gray A., Han C., Bisignano C., Rao P., Wool E. (2022). Global burden of bacterial antimicrobial resistance in 2019: A systematic analysis. Lancet.

[12] Naylor N.R., Atun R., Zhu N., Kulasabanathan K., Silva S., Chatterjee A., Knight G.M., Robotham J.V. (2018). Estimating the burden of antimicrobial resistance: A systematic literature review. Antimicrob. Resist. Infect. Control.

[13] Ayukekbong J.A., Ntemgwa M., Atabe A.N. (2017). The threat of antimicrobial resistance in developing countries: Causes and control strategies. Antimicrob. Resist. Infect. Control.

[14] Charani E., Smith I., Skodvin B., Perozziello A., Lucet J.-C., Lescure F.-X., Birgand G., Poda A., Ahmad R., Singh S. (2019). Investigating the cultural and contextual determinants of antimicrobial stewardship programmes across low-, middle- and high-income countries—A qualitative study. PLoS ONE.

[15] Dunachie S.J., Day N.P., Dolecek C. (2020). The challenges of estimating the human global burden of disease of antimicrobial resistant bacteria. Curr. Opin. Microbiol..

[16] Musoke D., Namata C., Lubega G.B., Niyongabo F., Gonza J., Chidziwisano K., Nalinya S., Nuwematsiko R., Morse T. (2021). The role of Environmental Health in preventing antimicrobial resistance in low- and middle-income countries. Environ. Health Prev. Med..

[17] Pezzani M.D., Tornimbene B., Pessoa-Silva C., de Kraker M., Rizzardo S., Salerno N.D., Harbarth S., Tacconelli E. (2021). Methodological quality of studies evaluating the burden of drug-resistant infections in humans due to the WHO Global Antimicrobial Resistance Surveillance System target bacteria. Clin. Microbiol. Infect..

[18] World Health Organization (WHO) Antimicrobial Resistance: Briefing to WHO Member States. March 2023. https://apps.who.int/gb/MSPI/pdf_files/2023/03/Item1_22-03.pdf.

[19] O’neill J.I.M. (2014). Antimicrobial resistance: Tackling a crisis for the health and wealth of nations. Rev. Antimicrob. Resist..

[20] Aponte-González J., González-Acuña A., Lopez J., Brown P., Eslava-Schmalbach J. (2019). Perceptions in the community about the use of antibiotics without a prescription: Exploring ideas behind this practice. Pharm. Pract..

[21] Wong L.P., Alias H., Husin S.A., Ali Z.B., Sim B., Ponnampalavanar S.S.L.S. (2021). Factors influencing inappropriate use of antibiotics: Findings from a nationwide survey of the general public in Malaysia. PLoS ONE.

[22] Aslam A., Gajdács M., Zin C.S., Ab Rahman N.S., Ahmed S.I., Zafar M.Z., Jamshed S. (2020). Evidence of the Practice of Self-Medication with Antibiotics among the Lay Public in Low- and Middle-Income Countries: A Scoping Review. Antibiotics.

[23] Opalska A., Kwa M., Leufkens H., Gardarsdottir H. (2020). Enabling appropriate use of antibiotics: Review of European Union procedures of harmonising product information, 2007 to 2020. Eurosurveillance.

[24] Abbo L.M., Cosgrove S.E., Pottinger P.S., Pereyra M., Sinkowitz-Cochran R., Srinivasan A., Webb D.J., Hooton T.M. (2013). Medical students’ perceptions and knowledge about antimicrobial stewardship: How are we educating our future prescribers?. Clin. Infect. Dis..

[25] Dyar O.J., Pulcini C., Howard P., Nathwani D. (2014). ESGAP (ESCMID Study Group for Antibiotic Policies). European medical students: A first multicentre study of knowledge, attitudes and perceptions of antibiotic prescribing and antibiotic resistance. J. Antimicrob. Chemother..

[26] Huang Y., Gu J., Zhang M., Ren Z., Yang W., Chen Y., Fu Y., Chen X., Cals J.W.L., Zhang F. (2013). Knowledge, attitude and practice of antibiotics: A questionnaire study among 2500 Chinese students. BMC Med. Educ..

[27] Khan A.K.A., Banu G., Reshma K.K. (2013). Antibiotic Resistance and Usage-A Survey on the Knowledge, Attitude, Perceptions and Practices among the Medical Students of a Southern Indian Teaching Hospital. J. Clin. Diagn. Res. JCDR.

[28] Minen M.T., Duquaine D., Marx M.A., Weiss D. (2010). A survey of knowledge, attitudes, and beliefs of medical students concerning antimicrobial use and resistance. Microb. Drug Resist..

[29] Scaioli G., Gualano M.R., Gili R., Masucci S., Bert F., Siliquini R. (2015). Antibiotic Use: A Cross-Sectional Survey Assessing the Knowledge, Attitudes and Practices amongst Students of a School of Medicine in Italy. PLoS ONE.

[30] Thriemer K., Katuala Y., Batoko B., Alworonga J.-P., Devlieger H., Van Geet C., Ngbonda D., Jacobs J. (2013). Antibiotic prescribing in DR Congo: A knowledge, attitude and practice survey among medical doctors and students. PLoS ONE.

[31] Wright E.P., Jain P. (2004). Survey of antibiotic knowledge amongst final year medical students. J. Antimicrob. Chemother..

[32] Bhardwaj K., Shenoy M.S., Baliga S., Unnikrishnan B., Baliga B.S. (2021). Knowledge, attitude, and practices related to antibiotic use and resistance among the general public of coastal south Karnataka, India—A cross-sectional survey. Clin. Epidemiol. Glob. Health.

[33] Pogurschi E.N., Petcu C.D., Mizeranschi A.E., Zugravu C.A., Cirnatu D., Pet I., Ghimpețeanu O.-M. (2022). Knowledge, Attitudes and Practices Regarding Antibiotic Use and Antibiotic Resistance: A Latent Class Analysis of a Romanian Population. Int. J. Environ. Res. Public Health.

[34] Shehadeh M., Suaifan G., Darwish R.M., Wazaify M., Zaru L., Alja’fari S. (2012). Knowledge, attitudes and behavior regarding antibiotics use and misuse among adults in the community of Jordan. A pilot study. Saudi Pharm. J..

[35] Sobeck J., Smith-Darden J., Gartner D., Kaljee L., Pieper B., Kilgore P., Zervos M. (2022). Antibiotic Knowledge, Beliefs, and Behaviors: Testing Competing Hypotheses Using an Urban Community Sample. Health Commun..

[36] Yu M., Zhao G., Stålsby Lundborg C., Zhu Y., Zhao Q., Xu B. (2014). Knowledge, attitudes, and practices of parents in rural China on the use of antibiotics in children: A cross-sectional study. BMC Infect. Dis..

[37] Nepal A., Hendrie D., Robinson S., Selvey L.A. (2019). Knowledge, attitudes and practices relating to antibiotic use among community members of the Rupandehi District in Nepal. BMC Public Health.

[38] Paredes J.L., Navarro R., Watanabe T., Morán F., Balmaceda M.P., Reateguí A., Elias R., Bardellini M., Ochoa T.J. (2022). Knowledge, attitudes and practices of parents towards antibiotic use in rural communities in Peru: A cross-sectional multicentre study. BMC Public Health.

[39] Alnasser A.H.A., Al-Tawfiq J.A., Ahmed H.A.A., Alqithami S.M.H., Alhaddad Z.M.A., Rabiah A.S.M., Albrahim M.A.A., Al Kalif M.S.H., Barry M., Temsah M.-H. (2021). Public Knowledge, Attitude and Practice towards Antibiotics Use and Antimicrobial Resistance in Saudi Arabia: A Web-Based Cross-Sectional Survey. J. Public Health Res..

[40] Napolitano F., Izzo M.T., Di Giuseppe G., Angelillo I.F. (2013). Public Knowledge, Attitudes, and Experience Regarding the Use of Antibiotics in Italy. PLoS ONE.

[41] Montalti M., Soldà G., Capodici A., Di Valerio Z., Gribaudo G., La Fauci G., Salussolia A., Scognamiglio F., Zannoner A., Gori D. (2022). Antimicrobial Resistance (AMR) in Italy over the Past Five Years: A Systematic Review. Biologics.

[42] Barchitta M., Quattrocchi A., Maugeri A., La Rosa M.C., La Mastra C., Sessa L., Cananzi P., Murolo G., Oteri A., Basile G. (2019). Antibiotic Consumption and Resistance during a 3-Year Period in Sicily, Southern Italy. Int. J. Environ. Res. Public Health.

[43] Sijbom M., Büchner F.L., Saadah N.H., Numans M.E., De Boer M.G.J. (2023). Trends in antibiotic selection pressure generated in primary care and their association with sentinel antimicrobial resistance patterns in Europe. J. Antimicrob. Chemother..

[44] ISTAT (2022). Bilancio Demografico Mensile e Popolazione Residente per Sesso, Anno 2022.

[45] Montuori P., Gioia M., Sorrentino M., Di Duca F., Pennino F., Messineo G., Maccauro M.L., Riello S., Trama U., Triassi M. (2023). Determinants Analysis Regarding Household Chemical Indoor Pollution. Toxics.

[46] Montuori P., Sorrentino M., Sarnacchiaro P., Di Duca F., Nardo A., Ferrante B., D’Angelo D., Di Sarno S., Pennino F., Masucci A. (2022). Job Satisfaction: Knowledge, Attitudes, and Practices Analysis in a Well-Educated Population. Int. J. Environ. Res. Public Health.

[47] Andrade C., Menon V., Ameen S., Kumar Praharaj S. (2020). Designing and conducting knowledge, attitude, and practice surveys in psychiatry: Practical guidance. Indian J. Psychol. Med..

[48] Khadka S., Hashmi F.K., Yadav G.K., Lamichhane S., Giri S., Tariq F., Amin S., Zaheer W., Akram K., Asghar I. (2023). Rational use of antimicrobials: A nationwide cross-sectional survey among people of Pakistan. Int. J. Surg. Glob. Health.

[49] Moon Z., Zuchowski M., Moss-Morris R., Hunter M.S., Norton S., Hughes L.D. (2022). Disparities in access to mobile devices and e-health literacy among breast cancer survivors. Support. Care Cancer.

[50] Özkan O., Özer Ö., Özmen S., Budak F. (2022). Investigation of the Perceived Coronavirus Threat, E-Health Literacy, and Psychological Well-Being in Turkey. Soc. Work Public Health.

[51] Shi Y., Ma D., Zhang J., Chen B. (2023). In the digital age: A systematic literature review of the e-health literacy and influencing factors among Chinese older adults. J. Public Health.

[52] Walters R., Leslie S.J., Polson R., Cusack T., Gorely T. (2020). Establishing the efficacy of interventions to improve health literacy and health behaviours: A systematic review. BMC Public Health.

[53] Blix H.S., Hjellvik V., Litleskare I., Rønning M., Tverdal A. (2011). Cigarette smoking and risk of subsequent use of antibacterials: A follow-up of 365,117 men and women. J. Antimicrob. Chemother..

[54] De Mello G.T., da Silva K.S., da Costa B.G., Borgatto A.F. (2019). Patterns of risk behaviors in Brazilian older adults: A latent class analysis. Geriatr. Gerontol. Int..

[55] Burnside C., Hudson T., Williams C., Lawson W., Laiyemo A.O. (2018). Sex differences in the use of healthcare services among US adults with and without a cancer diagnosis. Turk. J. Urol..

[56] Shebehe J., Ottertun E., Carlén K., Gustafson D. (2021). Knowledge about infections is associated with antibiotic use: Cross-sectional evidence from the health survey Northern Ireland. BMC Public Health.

[57] Marzan M., Islam D.Z., Lugova H., Krishnapillai A., Haque M., Islam S. (2021). Knowledge, Attitudes, and Practices of Antimicrobial Uses and Resistance Among Public University Students in Bangladesh. Infect. Drug Resist..

[58] Awad A.I., Aboud E.A. (2015). Knowledge, Attitude and Practice towards Antibiotic Use among the Public in Kuwait. PLoS ONE.

[59] Zucco R., Lavano F., Anfosso R., Bianco A., Pileggi C., Pavia M. (2018). Internet and social media use for antibiotic-related information seeking: Findings from a survey among adult population in Italy. Int. J. Med. Inform..

[60] Parveen S., Garzon-Orjuela N., Amin D., McHugh P., Vellinga A. (2022). Public health interventions to improve antimicrobial resistance awareness and behavioural change associated with antimicrobial use: A systematic review exploring the use of social media. Antibiotics.

[61] Waaseth M., Adan A., Røen I.L., Eriksen K., Stanojevic T., Halvorsen K.H., Garcia B.H., Holst L., Ulshagen K.M., Blix H.S. (2019). Knowledge of antibiotics and antibiotic resistance among Norwegian pharmacy customers—A cross-sectional study. BMC Public Health.

[62] Smith C.A., Chang E., Gallego G., Khan A., Armour M., Balneaves L.G. (2019). An education intervention to improve decision making and health literacy among older Australians: A randomised controlled trial. BMC Geriatr..

[63] Anderson A. (2018). Online health information and public knowledge, attitudes, and behaviours regarding antibiotics in the UK: Multiple regression analysis of Wellcome Monitor and Eurobarometer Data. PLoS ONE.

[64] Alejandro A.L., Bruce M., Leo C. (2022). Parents’ awareness of antimicrobial resistance: A qualitative study utilising the Health Belief Model in Perth, Western Australia. Aust. N. Z. J. Public Health.

[65] Higuita-Gutiérrez L.F., Roncancio Villamil G.E., Jiménez Quiceno J.N. (2020). Knowledge, attitude, and practice regarding antibiotic use and resistance among medical students in Colombia: A cross-sectional descriptive study. BMC Public Health.

[66] Gualano M.R., Gili R., Scaioli G., Bert F., Siliquini R. (2015). General population’s knowledge and attitudes about antibiotics: A systematic review and meta-analysis. Pharmacoepidemiol. Drug Saf..

[67] Read R.C., Cornaglia G., Kahlmeter G. (2011). Professional challenges and opportunities in clinical microbiology and infectious diseases in Europe. Lancet Infect. Dis..

[68] McNulty C.A.M., Lecky D.M., Farrell D., Kostkova P., Adriaenssens N., KoprivovaHerotova T., Holt J., Touboul P., Merakou K., Koncan R. (2011). Overview of e-Bug: An antibiotic and hygiene educational resource for schools. J. Antimicrob. Chemother..

